# Cell Signalling Pathways Leading to Novel Therapeutic Strategies in Cardiovascular Disease

**DOI:** 10.1155/2012/475094

**Published:** 2012-12-24

**Authors:** Sidney G. Shaw, David J. Abraham, Daryll M. Baker, Janice Tsui

**Affiliations:** ^1^Vasoactive Peptide Group, Department of Clinical Research, University of Bern, 3004 Bern, Switzerland; ^2^Centre for Rheumatology and Connective Tissues Diseases, Royal Free Hospital, Pond Street, London NW3 2QG, UK; ^3^Vascular Unit, Academic Department of Surgery, Royal Free Hospital, Pond Street, London NW3 2QG, UK; ^4^Division of Surgery & Interventional Science, University College London, Royal Free Campus Pond Street, London NW3 2QG, UK

## 1. Overview

Cardiovascular disease is a leading cause of morbidity and mortality with considerable financial impact worldwide. This special issue explores recent developments and novel strategies to target emerging risk factors central to the complex molecular mechanisms regulating the cardiovascular disease process. The concept was prompted by discussions arising from the UCL-Royal Free International Cardiovascular Diseases Workshops held each year at the Royal Free Hospital campus of University College London in collaboration with the University of Bern, Switzerland. These annual international workshops provide an integrated forum for clinicians and scientists involved in multidisciplinary studies in cardiovascular medicine to discuss recent advances aimed at integrating the latest findings in basic research more quickly and efficiently into medical practice.

The causes of cardiovascular disease are numerous but emerging areas of therapeutic interest are equally diverse and include novel molecular mechanisms by which cardiovascular risk factors and mediators, including reactive oxygen species and scavenger receptors, interact to regulate vascular cell proliferation and extra cellular matrix remodeling that play important roles during angiogenesis and cell response to injury and ischemia. 

The scavenger receptor LOX-1, for example, mediates OxLDL endocytosis via a clathrin-independent internalization pathway. Administration of LOX-1 antibodies in cellular and animal models suggests that such intervention inhibits atherosclerosis. Anti-atherogenic strategies that target LOX-1 function using gene therapy or small molecule inhibitors would be new ways to address the increasing incidence of vascular disease.

The role of special inflammatory molecules in the initiation and promotion of vascular inflammation is also under investigation. In particular the link between sterile inflammation, ischemia, and the innate immune system is providing important new information concerning the role of specific Toll-like receptors and endogenous molecules released in response to tissue injury. As sentinels of innate immunity TLRs are molecular pattern recognition receptors that recognize exogenous as well as tissue-derived molecular danger signals that promote chronic inflammation [1, 2]. Interestingly, these endogenously derived “danger” molecules appear to activate and induce differential TLR dimerisation compared to exogenously derived bacterial or viral components. Consequently modulation of TLR signaling by specific TLR agonists or antagonists, alone or in combination, may, therefore, be a novel therapeutic approach to treat various cardiovascular inflammatory conditions such as atherosclerosis, secondary microvascular complications of diabetes, and peripheral arterial disease (PAD) without compromising the normal immune response.

Additional signaling pathways and novel strategies that may prevent or slow the development of transplant arteriopathy and post ischemic reperfusion injury are likewise now beginning to be defined. Mechanisms by which haemodynamic forces such as pressure, stretch, and fluid shear stress are sensed by cells within the cardiovascular system are helping in the understanding of the atheroprotective benefits of steady laminar blood flow. 

Here, there is also evidence that nitric oxide (NO) and its endogenous inhibitor asymmetric dimethylarginine (ADMA) play significant roles. Recent experimental work implicates the ADMA-NO pathway and highlights the potential of manipulating this as a novel adjunct therapy. Related, mechanisms may be at play in relation to the damaging effects of oxidative stress on the development of enhanced fibroblast activity resulting in fibrosis of the skin, heart, and lungs, vascular dysfunction and ultimately internal organ failure, and death in scleroderma. Research suggests that the free radical nitric oxide (NO), a key mediator of oxidative stress, can profoundly influence the early microvasculopathy, and possibly the ensuing fibrogenic response. Recently, animal models and human studies have also identified dietary antioxidants, such as epigallocatechin-3-gallate (EGCG), which function as a protective system against oxidative stress and fibrosis. Hence, targeting EGCG may prove a possible candidate for therapeutic treatment aimed at reducing both oxidant stress and the fibrotic effects associated with scleroderma. 

Manipulation of cyclic GMP and cyclic AMP is a further emerging target for intervention in cardiovascular diseases such as hypertension, atherosclerosis, and heart failure. Drugs that enable the manipulation of these systems represent an exciting new area of pharmaceutical development.

Basic research has also identified a number of novel interventions that stimulate innate resistance of tissues to ischemia-reperfusion injury that may have important implications in the management of stroke and myocardial infarction. In particular, clinical trial data underpin one of these “conditioning” strategies, the phenomenon of remote ischemic preconditioning. This may in particular provide a novel cardioprotective strategy for the diabetic heart which evidence suggests may be more susceptible to ischemic reperfusion injury. 

Additionally, the study of connexins (Cxs) and cell-to-cell interactions via gap junctional communication is becoming an active area of investigation. Changes in Cxs found in diabetes are associated with both direct effects within the vasculature and indirect effects, by impairment of homeostasis in vital organs such as liver and kidney. Recent data suggests that Cxs targeting may alleviate some of the symptoms of microvascular complications, as demonstrated in recent work using topical Cx43 AsODN (antisense oligodeoxynucleotide) gel treatment. Cxs may also be used as future predictors of both diabetes progression and severity.

Similarly, erythropoietin (EPO) has tissue-protective properties, but increases the risk of thromboembolism by raising haemoglobin concentration. New generation EPO derivatives, however, are tissue protective without the haematopoietic side effects and preclinical studies have demonstrated their potential effectiveness and safety. 

Cytokine targeting may also play a future role in the therapy of peripheral vascular disease, in particular chemokine stromal-cell-derived factor-1 (SDF-1 aka CXCL12). Biological effects of SDF-1 are mediated by the chemokine receptor CXCR4, a 352-amino-acid rhodopsin-like transmembrane-specific G protein-coupled receptor (GPCR). There is evidence that the administration of SDF-1 increases blood flow and perfusion via recruitment of endothelial progenitor cells (EPCs).

As more becomes known, molecular genomic approaches are increasingly being used. MicroRNAs (miRNAs) are endogenous, small, noncoding RNAs that negatively control gene expression of target mRNAs. This phenomenon may be of benefit in regulating post-ischemic angiogenesis and vascular repair and analysis of circulating miRNAs may potentially be useful as potential biomarkers in ischemic diseases. Targeted *in situ* gene correction is currently being explored as a means to modulate Apolipoprotein E (ApoE) gene activity. APOE, a 34-kDa circulating glycoprotein, has pleiotropic anti-atherogenic functions and hence is a candidate to treat hypercholesterolaemia and atherosclerosis. In particular, we have the possibility and potential benefit of combining two technological advances to repair aberrant APOE genes: (i) an engineered endonuclease to introduce a double-strand break in exon 4, which contains the common, but dysfunctional, *ε*2 and *ε*4 alleles; (ii) an efficient and selectable template for homologous recombination (HR) repair, namely, an adeno-associated viral (AAV) vector, which harbors the wild-type APOE sequence. This technology is currently applicable *ex vivo*, for example, to target haematopoietic or induced pluripotent stem cells, and also for *in vivo* organ gene targeting. It is to be hoped that such emerging technology will eventually translate into patient therapy to reduce CVD risk.

Other genome-wide association studies (GWAS) have revolutionized research into genetic variants that underpin the development of many complex diseases including abdominal aortic aneurysm and allowed the exploration of mechanisms by which identified loci may contribute to its development. Studies have also highlighted the potential of post-GWAS analytical strategies to improve our understanding of the disease further.

Finally, progress in the identification of key mechanisms regulating tissue repair, and the potential of stem cell therapy and tissue engineering, offer promising new possibilities in bypass grafting and recovery from cardiac injury. 

We hope that readers will find that this special issue addresses the important challenges, opportunities, and recent developments that are currently being exploited in cardiovascular research with the potential to promote the implementation of novel translational strategies for optimizing the care and treatment of patients with cardiovascular disease. 
